# Terminal nucleotidyltransferase *Tent2* microRNA A-tailing enzyme regulates excitatory/inhibitory balance in the hippocampus

**DOI:** 10.1261/rna.080240.124

**Published:** 2025-06

**Authors:** Patrycja Wardaszka-Pianka, Bozena Kuzniewska, Natalia GumiNska, Anna Hojka-Osinska, Monika Puchalska, Jacek Milek, Aleksandra Stawikowska, Pawel Krawczyk, Francois P. Pauzin, Tomasz Wojtowicz, Kasia Radwanska, Clive R. Bramham, Andrzej Dziembowski, Magdalena Dziembowska

**Affiliations:** 1 Department of Animal Physiology, Faculty of Biology, University of Warsaw, 02-096 Warsaw, Poland; 2 Laboratory of RNA Biology, International Institute of Molecular and Cell Biology, 02-109 Warsaw, Poland; 3 Bioinformatics Facility, International Institute of Molecular and Cell Biology, 02-109 Warsaw, Poland; 4 Laboratory of Molecular Basis of Behavior, Nencki Institute of Experimental Biology, 02-093 Warsaw, Poland; 5 Department of Biomedicine, University of Bergen, 5007 Bergen, Norway; 6 Mohn Research Center for the Brain, University of Bergen, 5007 Bergen, Norway; 7 Laboratory of Cell Biophysics, Nencki Institute of Experimental Biology, 02-093 Warsaw, Poland

**Keywords:** *Tent2*, neuron, miRNA, mRNA poly(A) tail, excitatory/inhibitory balance

## Abstract

One of the posttranscriptional mechanisms regulating the stability of RNA molecules involves the addition of nontemplated nucleotides to their 3′ ends, a process known as RNA tailing. To systematically investigate the physiological consequences of terminal nucleotidyltransferase TENT2 absence on RNA 3′ end modifications in the mouse hippocampus, we developed a new *Tent2* knockout mouse. Electrophysiological measurements revealed increased excitability in *Tent2* KO hippocampal neurons, and behavioral analyses showed decreased anxiety and improved fear extinction in these mice. At the molecular level, we observed changes in miRNAs’ monoadenylation in *Tent2* KO mouse hippocampus, but found no effect of the TENT2 loss on the mRNAs’ total poly(A) tail length, as measured by direct nanopore RNA sequencing. Moreover, differential expression analysis revealed transcripts related to synaptic transmission to be downregulated in the hippocampus of *Tent2* knockout mice. These changes may explain the observed behavioral and electrophysiological alterations. Our data thus establish a link between TENT2-dependent miRNA tailing and the balance of inhibitory and excitatory neurotransmission.

## INTRODUCTION

The mRNA poly(A) tails added by canonical nuclear poly(A) polymerase (PAP) in the nucleus are vital for regulating mRNA stability, export to the cytoplasm, translation, and decay. In addition to classical poly(A) adenylation, the 3′ ends of various classes of RNAs are modified by enzymes known as terminal nucleotidyltransferases (TENTs), which add nontemplated nucleotides to their 3′ ends, a process known as RNA tailing. These posttranscriptional modifications affect RNA stability and function and were shown to be crucial in early embryonic development and gamete production. Cytoplasmic polyadenylation is best known from studies of gametogenesis (Wang et al. 2002; Kim and Richter 2006; Kim et al. 2010; Sartain et al. 2011) where specific mRNAs are rapidly polyadenylated in response to cellular signals, allowing translation to start in a transcription-independent fashion.

In humans, there are 11 different TENTs. TENT2 (terminal nucleotidyltransferase 2, GLD2) is a noncanonical PAP, that adds adenosine nucleotides to the 3′ end of RNAs. TENT2 was first described in nonmammalian species, including *Ceanorhabditis elegans*, *Xenopus laevis*, and *Drosophila melanogaster*. In these organisms, TENT2 was shown to elongate poly(A) tails of a subset of stored cytoplasmic mRNAs, thus regulating their translational activation in early development (Wang et al. 2002; Barnard et al. 2004; Rouhana et al. 2005; Benoit et al. 2008; Cui et al. 2008, 2013; Nousch et al. 2017). Overall, the studies on nonmammalian species seemed to show that cytoplasmic polyadenylation, is a major regulatory mechanism during oocyte maturation and egg activation conserved between species.

However, in mice, *Tent2* disruption did not affect the maturation of the oocytes or animals’ fertility (Nakanishi et al. 2007), nor the poly(A) tail elongation in oocytes using reporter RNAs. *Tent2*-deficient mice remain fertile and healthy. At the same time, another family of PAPs, TENT5s, plays crucial roles in mammalian oogenesis and spermatogenesis (Brouze et al. 2024). However, TENT2 is widely expressed in the mouse brain, where it can regulate posttranscriptional modifications of RNAs involved in synaptic plasticity. TENT2 was suggested to polyadenylate specific target mRNAs, such as NR2A subunit of *N*-methyl-d-aspartate receptor (Udagawa et al. 2012), essential for long-term synaptic plasticity. However, *Tent2* knockout (KO) mice generated with a classical ES call-based approach do not display any obvious behavioral abnormalities (Mansur et al. 2016).

In addition to its role in mRNA expression, TENT2 was shown to regulate the stability of certain mature miRNAs, and its deletion in a human fibroblast cell line significantly reduced the fraction of tailed miRNAs possessing a nontemplated monoadenosine (D'Ambrogio et al. 2012). Furthermore, the liver-specific miRNA-122, is 3′ monoadenylated in human hepatocytes and mouse liver (Katoh et al. 2009). Consistently, *Tent2* KO mice show significantly lower levels of miRNA-122, indicating that TENT2 monoadenylation enhances its stability (Katoh et al. 2009).

Knowing that TENT2 is ubiquitously expressed in the nervous system, in this study, we generated a *Tent2* KO mouse line using CRISPR/Cas9 to revisit the potential role of this enzyme in neuronal RNA metabolism and physiology. Our data indicate that TENT2 is involved in the monoadenylation of miRNAs in mouse hippocampus as we observed changes in the nontemplated isomiRs populations. On the other hand, we found no effect of the TENT2 loss on the total poly(A) tail length of mRNAs, as measured by direct RNA sequencing (DRS) (Krawczyk et al. 2024). The analysis of changes in expression profiles of altered mono(A)-tailed miRNAs and their target mRNAs did not reveal correlation. However, among the dysregulated mRNAs, we identified a group of transcripts related to neurotransmitter transport and synaptic transmission that were downregulated in the hippocampus of *Tent2* KO mice. The electrophysiological measurements revealed increased excitability of *Tent2* KO neurons, and the analyses of mice behavior have shown decreased anxiety and improvement in fear extinction of *Tent2* KO mice. Our data establish a link between TENT2-dependent microRNA tailing and the balance of inhibitory and excitatory neurotransmission.

## RESULTS AND DISCUSSION

**FIGURE 1.**
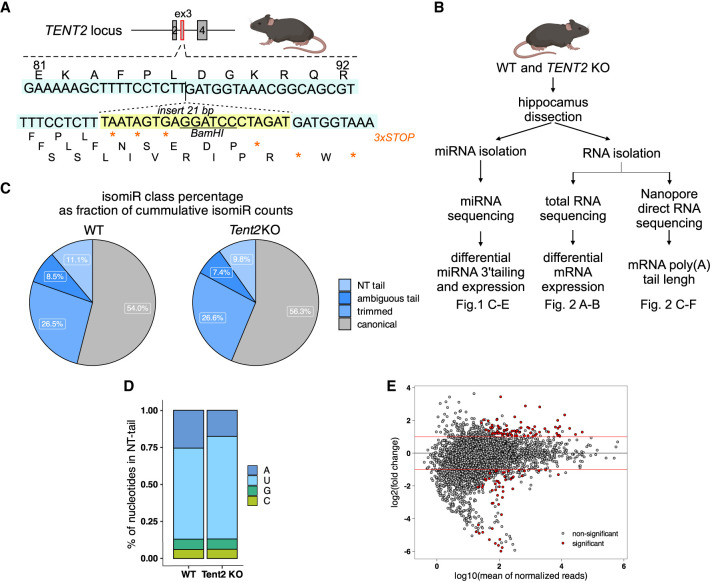
miRNA sequencing and poly(A) tail length analysis in the hippocampus of *Tent2* KO mice. (*A*) Schematic illustration of *Tent2* locus fragment. Position of the 21 bp insert is indicated; the sequence is marked with a yellow box. To facilitate the detection of mutations, a restriction site for BamHI was introduced. STOP codons introduced in the insert are marked with asterisks. (*B*) Workflow of the experiments presented in panels *C*–*E* and on Figure 2, depicting isolation of RNA from the hippocampi and different sequencing approaches. (*C–E*) Results of miRNA sequencing of *Tent2* KO and WT hippocampi (WT *n* = 3, *Tent2* KO *n* = 5). (*C*) IsomiR class percentage analysis, showing that 11.1% of isomiRs in WT and 9.8% in *Tent2* KO had NT tail at the 3′ end. (*D*) Significant reduction of adenylation accompanied by an increase in uridylation in the NT tails in *Tent2* KO hippocampi. (*E*) miRNA abundance in *Tent2* KO hippocampi when compared to WT.

To assess the role of TENT2 PAP in the brain, we generated a new *Tent2* KO mouse line using genome editing technology (Fig. 1A). The homozygous *Tent2* KO mice displayed no visibly harmful phenotype, were viable and fertile, and produced similar litter sizes to wild-type (WT) mice. To elucidate the consequences of the absence of TENT2 enzymatic activity on RNA 3′ end modifications in the mouse hippocampus, we conducted: (i) microRNA sequencing, (ii) poly(A) tail length analysis using nanopore DRS, and (iii) total mRNA sequencing with Illumina (Fig. 1B).

### TENT2-dependent adenylation of miRNA does not affect their stability in mouse hippocampus

In the previous study of Mansur et al., the authors have shown a reduction in the amount of monoadenylation of miRNAs, but not in the miRNA levels themselves in the hippocampus of another *Tent2* KO mouse model (Mansur et al. 2016). To corroborate this study in our *Tent2* KO mice and further correlate the results with mRNA polyadenylation status, we performed small RNA sequencing (sRNA-seq). We generated the sRNA libraries using hippocampi from five *Tent2* KO and three WT male mice (littermates). As our focus was the 3′ nontemplated additions to the miRNAs, we analyzed miRNA expression levels and 3′ miRNA isoforms (isomiRs) composition using QuagmiR (Bofill-De Ros et al. 2019). It is a highly customizable tool for comprehensive analysis of heterogeneous isomiRs populations and allows for various 5′ and 3′ templated and not templated (NT), tailed as well as trimmed isomiRs detection. We consistently detected 3452 isomiRs resulting from 576 miRNAs in all eight analyzed samples. The primary analysis showed that >50% of analyzed reads were assigned to the canonical form of miRNA (annotated mature miRNA sequence). Then, on average, 26.5% of reads represented trimmed miRNA isoforms, and the remaining were tailed isomiRs. According to our results, on average, only 10% of isomiRs had an NT tail at their 3′ end. Even if the NT-tailed isomiRs did not constitute the majority of miRNA populations, we still could observe a slight decrease in their accumulation under *Tent2* KO (change from 11.1% in WT samples on average to 9.8% on average in *Tent2* KO samples) (Fig. 1C; Supplemental Fig. S1A). Notably, adenosine frequency was significantly reduced in the NT tails under *Tent2* KO (Fig. 1D; Supplemental Fig. S1B). Moreover, the decreased adenylation was accompanied by a moderate increase in uridylation.

Further, we analyzed NT tails in miRNAs by their length and composition. Intriguingly, we did not observe a significant decrease in global 1 nucleotide (nt) additions but only in the 2 and 3 nt additions (Supplemental Fig. S1C). This was explained when the changes in tail composition by length were examined. *Tent2* KO led to a reduction of accumulation of any type of tail that contained adenosines. The decrease of mono(A) tails was compensated by the mono(U) and mono(C) additions. For the 2 and 3 nt tails, we observed a prominent drop in the accumulation of tails containing adenosines.

To explore the impact of reduced monoadenylation on miRNA abundance, we compared the levels of all isomiRs in *Tent2* KO hippocampi with the WT. The analysis revealed that more than 7% of isomiRs were significantly dysregulated in *Tent2* KO, with a similar number of upregulated and downregulated ones (Fig. 1E; Supplemental Data 2). The differential analysis confirmed our observations of changes in tail composition, as the pattern of drop of many isomiRs under the *Tent2* KO is mainly due to decreased accumulation of isomiRs with tails enriched in adenosines (Supplemental Fig. S1D). As the tailed isomiRs are not the main form of miRNA expressed, we examined how the change in accumulation of mono(A) tail isomiRs (as an example of TENT2 action) correlates with the accumulation of canonical form. This analysis showed no correlation between the changes in 3′ monoadenylation and the alteration in abundance of canonical miRNA (Supplemental Fig. S1E). The fact that both upregulated and downregulated canonical miRNAs were observed in *Tent2* KO samples when the mono(A)-tailed form of miRNAs was decreased indicates that the underlying mechanisms are complex. Together, these results point to TENT2 as an enzyme responsible for the adenylation of miRNAs in mouse hippocampus. However, since the changes in tailed isomiRs under *Tent2* KO are rather minor and do not disturb globally canonical miRNA accumulation profiles, it seems that TENT2 is not the only enzyme regulating 3′-end RNA metabolism.

### *Tent2* knockout mice show changes in the expression of mRNAs related to neurotransmission but no significant differences in mRNA poly(A) tail length

In addition to miRNA analysis, the RNA samples isolated from *Tent2* KO and WT hippocampi were processed using two high-throughput platforms. The mRNA expression levels were determined by total RNA sequencing with Illumina (RNA-seq), whereas the poly(A) tail lengths were assessed at the whole transcriptome scale by Oxford Nanopore DRS (Fig. 1B).

**FIGURE 2.**
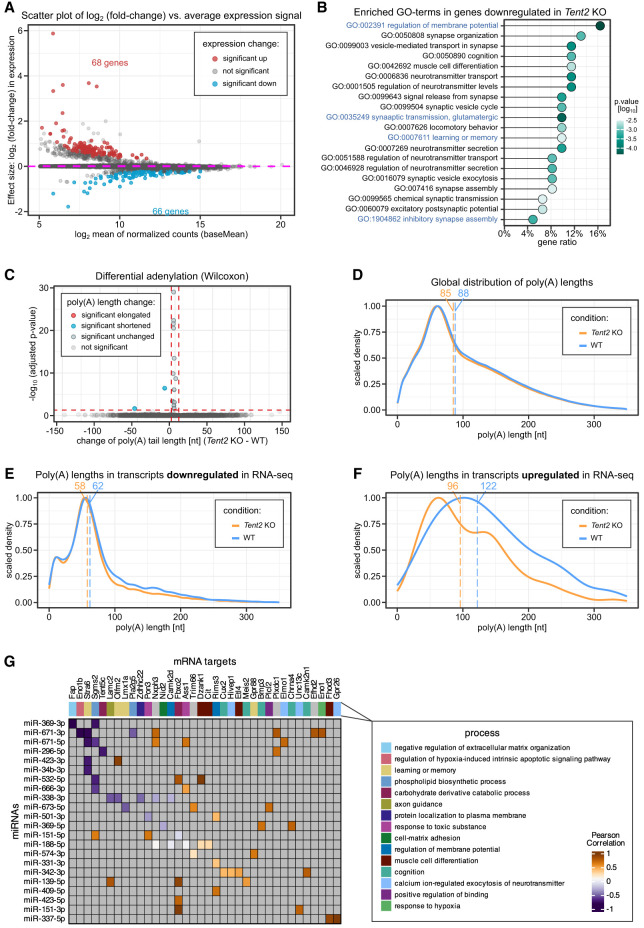
Results of RNA sequencing of mRNAs in the hippocampus of *Tent2* KO and WT mice. (*A*) Differential expression of mRNAs in *Tent2* KO hippocampi versus WT (*n* = 3/genotype). Genes with significantly changed expression are highlighted (red, upregulated; blue, downregulated). The data were shrunken for clarity. (*B*) Gene ontology analysis of transcripts downregulated in *Tent2* KO mice. (*C*) Differential adenylation analysis between *Tent2* KO and WT mice. Dashed lines indicate significance cutoff values. (*D*) Global distribution of poly(A) tail lengths of mRNAs in *Tent2* KO and WT mice (*n* = 3 mice/genotype). (*E*) Distribution of poly(A) tail lengths in mRNAs with expression downregulated in RNA-seq. (*F*) Distribution of poly(A) tail lengths in mRNAs with expression downregulated in RNA-seq. For panels *D*–*F*, dashed lines indicate median values (also provided *above* the plotting area). (*G*) Pearson's correlation analyses on mRNA and miRNA expression levels. Analysis included mono(A)-tailed isomiRs with predicted binding sites in genes that were significantly deregulated under Tent2 KO. Violet, inverse correlation; brown, positive correlation. A distinct gene assigned to a potential biological process that they regulate.

Differential expression analysis of the RNA-seq data identified 134 differentially expressed genes: 66 downregulated and 68 upregulated (Fig. 2A; Supplemental Data 3). Notably, a majority of the upregulated genes (44) are annotated in the reference transcriptome as putative genes, lncRNAs or pseudogenes (e.g., Gm4735, 1700080N15Rik). Many of them have only a temporary name and yet unverified function. The remaining upregulated protein-coding genes were not recognized as having a direct link to strictly neuronal functions and synaptic connectivity. In contrast, all the mRNAs downregulated in *Tent2* KO mice correspond to well-characterized, reliable protein-coding genes. Among them, we noticed a significant overrepresentation of mRNAs involved in the regulation of membrane potential, neurotransmitter transport, glutamatergic synaptic transmission, learning and memory, and inhibition of synaptic transmission (Fig. 2B). We then examined the poly(A) tail lengths of the DRS data. Interestingly, we found no global effect in poly(A) tail length distributions across the phenotypes studied (Fig. 2C,D). For downregulated mRNAs, we observed a subtle, albeit not statistically significant, shortening in poly(A) tail lengths between genotypes (median lengths of 58 in KO vs. 62 in WT; Fig. 2E). More pronounced differences in poly(A) tail length distributions were noted in upregulated genes, though these differences were also not statistically significant (median lengths of 96 in KO vs. 122 in WT; Fig. 2F).

Next, we investigated the correlation of expression profiles between miRNAs and target mRNAs in search of any possible consequences of observed minor NT-tailed isomiR changes on the mRNA accumulation. For the identification of miRNA:target pairs, a list of 158 mono(A)-tailed isomiRs and a list of pairs of microRNAs and their target ensemble IDs obtained from miRBase were used. The target identification was successful for 23 miRNAs with the mono(A)-tailed isoform, which, according to predictions, take part in 15,460 interactions with 9269 genes. For the inspection of expression profiles correlation, we focused only on differentially accumulated mRNAs and analyzed 61 interactions with 22 miRNAs and 36 genes (Fig. 2G; Supplemental Fig. S2A,B; Supplemental Data 4). Our results show that the percentage of miRNA:target interactions having a negative correlation in expression profiles is lower than the percentage of interactions having a positive correlation (36% and 67%, respectively). However, miRNA:target mRNA interactions that are involved in the repression of protein synthesis may not result from considerable dysregulation of transcripts accumulation levels; thus, sometimes uncorrelated expression levels may also be observed. Profound analysis of miRNA:target pairs revealed their involvement in processes such as learning and memory, regulation of membrane potential, axon guidance, or protein localization to the plasma membrane (Fig. 2G; Supplemental Fig. S2B).

Among the transcripts interacting with mono(A)-tailed isomiRs, *Stra6* mRNA has been paired with four different miRNAs. Stra6 encodes a receptor for retinol uptake that is crucial for the homeostasis of vitamin A and retinoic acid. Retinoic acid is synthesized during synaptic inactivity, and it induces both: the insertion of GluA2-lacking calcium-permeable AMPA receptors (Chen et al. 2012) and the internalization of synaptic GABAA receptors (Sarti et al. 2013). Thus, its action leads to increased excitatory and decreased inhibitory transmission. The other identified transcript paired with several miRNAs is *Sgms2* (sphingomyelin synthase). It has been reported that *Sgms2* KO mice are impaired in the Morris water maze (Wang et al. 2017), indicating an alteration in hippocampal spatial learning and memory. Among other miRNA:target pairs, we identified mRNAs involved in the regulation of synaptic transmission, such as *Rims3*, that interacts with presynaptic voltage-dependent Ca^2+^ channels (VDCCs) and increases neurotransmitter release (Takada et al. 2015) and *Unc13c* (*Munc13-3*), that mediates synaptic vesicle priming and regulates short-term synaptic plasticity (Lipstein et al. 2012) and plays a critical role in the formation of release sites with calcium channel nanodomains (Kusch et al. 2018).

Finally, knowing that TENT2 regulates the selection of polyadenylation sites (PAS) (Mansur et al. 2021) and that the length of the 3′ UTR regulates the accessibility of effectors such as miRNAs to their binding sites (Diener et al. 2024), we investigated the occurrence of mRNA isoforms exploiting alternative PAS. Our analysis revealed that polyadenylation site choice does not correlate with differential expression. Furthermore, *Tent2* KO showed no consistent trend, with a nearly equal number of genes exhibiting 3′-UTR elongation (distal site usage; 59 genes) and shortening (proximal site usage; 60 genes) (Supplemental Fig. S2C,D). For PAS within the last intron, there was a slight preference for distal site usage (86 genes) over proximal site usage (62 genes). However, no association was observed between miRNA interactions and polyadenylation site selection. Only individual genes—*Cux2* (3′ UTR) and *Camk2d* (intron sites)—showed both miRNA interaction and distal site preference (Supplemental Fig. S2D). While these genes are implicated in neuroplasticity, our findings suggest that polyadenylation site selection is not a central mechanism driving the *Tent5* KO phenotype.

Altogether, the investigations of molecular characteristics of *Tent2* KO in mouse hippocampus showed dysregulation of some transcripts; however, the underlying mechanism is rather complex and could not be explained with the changes in the 3′-end processing.

### Increased excitability and decreased inhibitory synaptic transmission in *Tent2* KO hippocampal neurons

**FIGURE 3.**
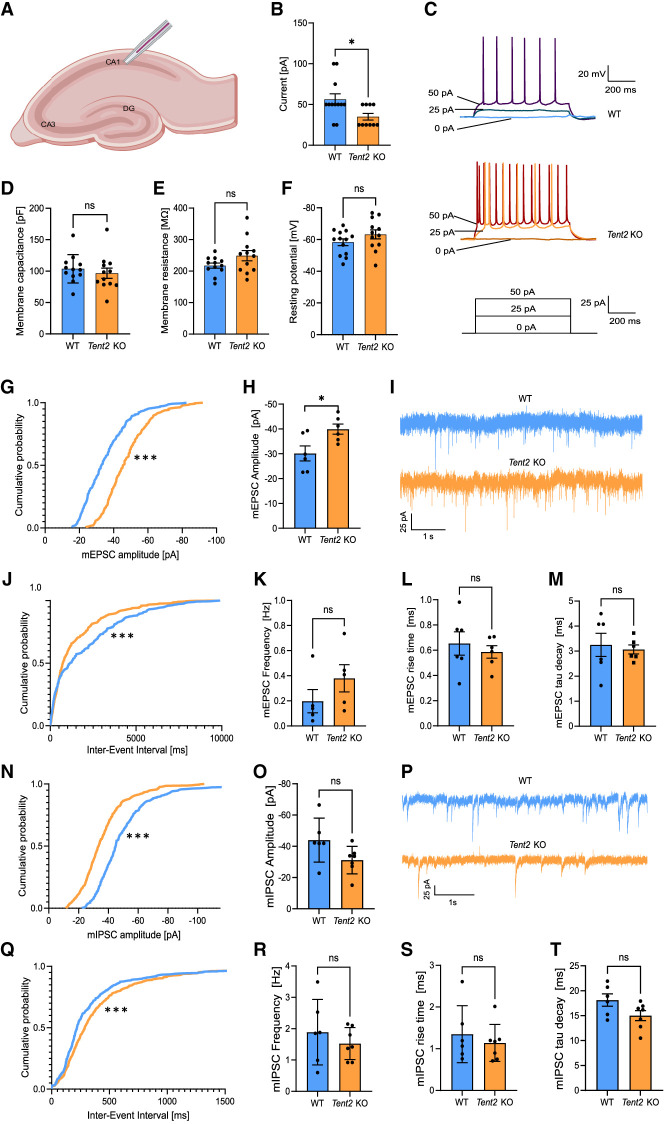
*Tent2* KO neurons exhibit increased excitability in CA1 in hippocampal slices (*A*–*F*) and enhanced excitatory synaptic transmission in cultured hippocampal neurons (*G*–*M*). (*A*) Schematic of patch-clamp recordings in CA1 region of hippocampal horizontal slices. (*B*) Rheobase, the lowest value of current to trigger an action potential was significantly lower for *Tent2* KO (Mann–Whitney test; [*] *P* = 0.0257). (*C*) Representative voltage responses to current stimuli for WT (*top* panel) and *Tent2* KO neurons (*middle* panel). (*D–F*) Statistics for selected passive membrane properties (see Materials and Methods for description). Membrane capacitance: membrane resistance or resting membrane potential were not altered in *Tent2* KO neurons. Data were obtained from 12 to 13 neurons per group. The data are expressed as mean ± SEM. (*G*–*M*) AMPA/kainate receptor glutamatergic transmission was recorded in 21–22 DIV hippocampal cultures with whole-cell patch-clamp method. (*G*) The cumulative probability of individual mEPSCs amplitudes was shifted toward higher values (Kolmogorov–Smirnov test; *P* < 0.00001). (*H*) Averaged amplitude of mEPSCs was significantly larger for *Tent2* KO neurons (*t*-test; *P* < 0.05). (*I*) Representative current traces from WT and *Tent2* KO neurons. (*J*) The cumulative distribution function for inter-event time intervals between subsequent mEPSCs is left-shifted compared to WT neurons (Kolmogorov–Smirnov test *P* < 0.0001). (*K*) The averaged frequency of mEPSCs is not significantly changed between genotypes. (*L* and *M*) *Tent2* KO neurons do not exhibit altered mEPSCs rise time (*L*) or tau decay (*M*). Data were obtained from six neurons per group. The data are expressed as mean ± SEM. (*N*–*T*) Reduction of inhibitory synaptic transmission in *Tent2* KO cultured hippocampal neurons. (*N*) The cumulative probability of amplitudes for mIPSCs is left-shifted (Kolmogorov–Smirnov test; *P* < 0.0001). (*O*) Averaged amplitudes of mIPSCs were not significantly different in *Tent2* KO neurons (*t*-test; *P* = 0.07). (*P*) Representative current traces of mIPSCs recordings from WT and *Tent2* KO cultured neurons (21–22 DIV). (*Q*) The cumulative probability plot of the inter-event interval for *Tent2* KO is right-shifted relative to the WT neurons (Kolmogorov–Smirnov test *P* < 0.0001). (*R*) The averaged frequency of mIPSCs was not significantly changed. (*S* and *T*) mIPSCs rise time (*S*) and tau decay (*T*) were not altered in *Tent2* KO neurons. Data were obtained from six to seven neurons per group. The data are expressed as mean ± SEM. Partially created with Biorender.

Among the mRNAs differentially expressed in the *Tent2* KO hippocampus, we identified an overrepresentation of transcripts involved in the regulation of neurotransmitter levels and synaptic transmission. Also, the analysis of miRNA:target pairs revealed involvement in learning and memory as well as regulation of membrane potential. We, therefore, aimed to assess the electrophysiological properties of neurons from *Tent2* KO using patch-clamp. Recordings were performed in acute brain slices prepared from six WT and four *Tent2* KO mice. We measured passive membrane properties and membrane excitability of pyramidal neurons in the CA1 hippocampal region (Fig. 3A). Rheobase, the minimal current magnitude that evoked an action potential, was significantly decreased in *Tent2* KOs (Fig. 3B,C; Mann–Whitney test; [*] *P* = 0.0257). The majority of *Tent2* KO neurons generated an action potential at 25 pA of applied current, whereas most of the WT neurons started firing at 50 pA. First spike latency (Supplemental Fig. S3A), general frequency of spiking (Supplemental Fig. S3B), and accommodation ratio (Supplemental Fig. S3C) remained unchanged but the frequency of the spikes during the first half of the stimulus pulse was decreased for *Tent2* KO neurons at final current values. We did not observe any significant differences in membrane capacitance (Fig. 3D), membrane resistance (Fig. 3E), or resting membrane potential (Fig. 3F) between *Tent2* KO and WT neurons. Altogether, *Tent2* KO neurons showed increased excitability in response to current stimuli without a change in the key passive membrane properties or kinetics of individual action potentials.

Increased excitability of pyramidal cells in hippocampal CA1 may be due to enhanced excitatory or reduced inhibitory synaptic transmission. To verify which one is disturbed, we measured both miniature excitatory and inhibitory currents with the whole-cell patch-clamp method. To this end, we chose to study cultured neurons, which exhibit a significant amount of synaptic events compared to the acute brain slices model. First, we measured miniature excitatory postsynaptic currents (mEPSCs) in 21–22 DIV-cultured primary hippocampal neurons. In *Tent2* KO neurons, the amplitude of mEPSCs was increased, as shown in the cumulative probability of amplitudes for individually recorded miniature currents (Fig. 3G; Kolmogorov–Smirnov test; *P* < 0.00001) and the averaged mEPSCs amplitudes (Fig. 3H; *t*-test; *P* < 0.05). The representative traces (Fig. 3I) suggested increased mEPSC frequency, and this was corroborated by the cumulative distribution function showing a significantly shorter mEPSC inter-event interval in *Tent2* KO neurons relative to WT (Fig. 3J; Kolmogorov–Smirnov test; *P* < 0.0001). In addition, averaged mEPSCs frequency (Fig. 3K) was almost two times higher in *Tent2* KO neurons, although this change was not statistically significant. The rise-time (Fig. 3L) and tau decay (Fig. 3M) of the currents were also not significantly different between genotypes. Thus, cultured *Tent2* KO neurons exhibited enhanced excitatory synaptic transmission. These results confirm the results obtained with acute brain slices.

Next, we recorded GABA_A_ receptor-mediated miniature inhibitory postsynaptic currents (mIPSCs) in 21–22 DIV cultured primary hippocampal neurons. The comparison of cumulative probability of mIPSCs amplitude between WT and *Tent2* KO (Fig. 3N) neurons showed that mIPSCs amplitudes are smaller in *Tent2* KO neurons (Kolmogorov–Smirnov test; [***] *P* < 0.0001). Averaged amplitudes (Fig. 3O) demonstrated the same trend, but the difference was not statistically significant (*t*-test; *P* = 0.07). The intervals between subsequent mIPSCs (Fig. 3Q) was elongated in *Tent2* KO neurons (Kolmogorov–Smirnov test; [***] *P* < 0.0001). However, there was no visible reduction of the average mIPSC frequency (Fig. 3R).

Altogether, electrophysiological measurements in hippocampal neurons indicated that *Tent2* KO neurons may exhibit increased excitability. In addition, when *Tent2* KO neurons are cultured, they exhibit enhanced excitatory and decreased inhibitory synaptic transmission. Therefore, we have next examined whether observed alterations in excitatory/inhibitory (E/I) balance following *Tent2* KO may be associated with altered behavior in mice.

### Decreased anxiety and increased fear memory extinction in *Tent2* KO mice

**FIGURE 4.**
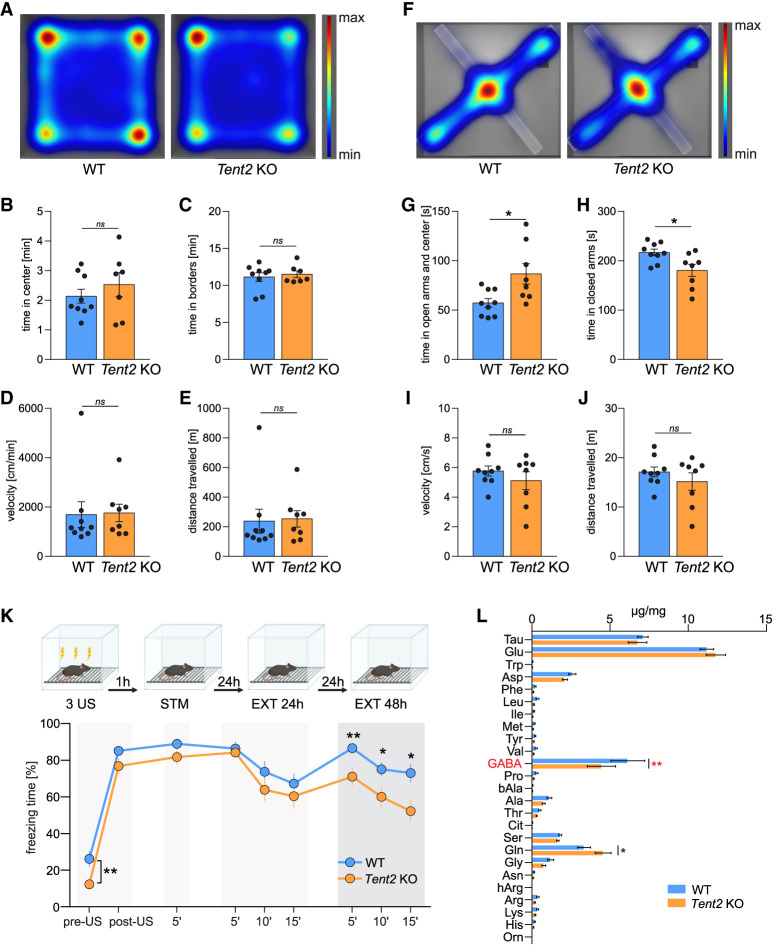
Behavioral characterization of *Tent2* KO and WT mice. Anxiety, exploratory behavior, and locomotion were assessed using open field (*A*–*E*) and elevated plus maze (*F*–*J*) tasks. Learning and memory were assessed using contextual fear conditioning (CFC) (*K*). (*A*) Population-averaged heatmaps showing mice activity in the open arena. No significant differences in time spent in the center (*B*), time spent in borders (*C*), velocity (*D*), or distance moved (*E*) were observed (*P*-value >0.05, unpaired *t*-test, *n* = 8–9). (*F*) Population averaged heatmaps showing mice activity in the elevated plus maze. *Tent2* KO mice spent significantly more time in the open arms and in the center as compared to WTs (*G*, [*] *P*-value = 0.0156, unpaired *t*-test, *n* = 8–9) and significantly less time in the closed arms (*H*, *P*-value = 0.017, unpaired *t*-test, *n* = 8–9). Similarly, as in the open field task, no significant differences in velocity (*I*) or distance traveled (*J*) were observed (*P*-value >0.05, unpaired *t*-test, *n* = 8–9). (*K*) Experimental timeline (*upper* panel) and summary of data showing freezing levels during CFC and test sessions. Mice were conditioned in a novel context (3US presentations, 2 sec, 0.7 mA). Next, the mice were re-exposed three times to the same context: 1 h after CFC to assess short-term contextual fear memory (STM, 5 min); 24 h after CFC to test long-term contextual fear memory and to extinguish contextual fear (EXT1, 15 min); and 48 h after CFC to test fear extinction memory (EXT2, 15 min). *Tent2* KO mice showed increased locomotion before the training (pre-US) as compared to WT animals ([**] *P*-value = 0.01), but did not differ in freezing behavior post-US (*P*-value >0.05), 1 h after training (STM) nor 24 h after training (LTM). Interestingly, *Tent2* KO mice showed decreased freezing levels during EXT2 ([**] *P*-value = 0.009, [*] *P*-value = 0.039, [*] *P*-value = 0.012). Data are presented as mean ± SEM; *n* = 18–22; two-way ANOVA, post hoc Sidak's multiple comparisons test. (*L*) Levels of amino acids in the hippocampi of fear-conditioned *Tent2* KO and WT mice. Directly after the last test in the CFC experiment shown in panel *K*, mice were sacrificed and the levels of amino acids were measured using mass spectrometry. Significantly decreased levels of GABA neurotransmitter were detected in the hippocampi of *Tent2* KO mice as compared to WT ([**] *P*-value = 0.0038). Additionally, increased levels of glutamine were observed in *Tent2* KO hippocampi ([*] *P*-value = 0.0267). Data are presented as mean ± SEM; *n* = 8–9; two-way ANOVA, post hoc Sidak's multiple comparisons test.

We performed behavioral tests to assess anxiety, locomotor activity as well as learning and memory. Firstly, we tested the mice in the open field task (Fig. 4A–E). We did not observe any significant differences in the locomotor activity (velocity and distance traveled, Fig. 4D,E) of *Tent2* KO and WT mice. Similarly, we did not detect any differences in the marble burying test (Supplemental Fig. S4), that is used to study compulsive-like behaviors. In contrast, we observed increased exploration of open arms and decreased time spent in closed arms of elevated plus maze by *Tent2* KO mice (Fig. 4F,J), indicating decreased anxiety. Similarly, as in the previous tests, no significant differences in the locomotor activity were observed (Fig. 4I,J). Our data are consistent with the results of Mansur et al. (2016) where in another *Tent2* KO model no behavioral differences in the open field and marble burying tests were seen. One significant difference in our study was the decreased anxiety of Tent2 KO mice in an elevated plus maze, which was not observed by Mansur and colleagues. The observed discrepancy may be due to the use of a different mouse line, developed using a distinct gene-targeting methodology and genetic background: previously C57BL/6 inbred mouse strain (Mansur et al. 2016) versus F1 hybrid (C57BL/6 × CBA)Tar mixed background in the current paper. It has been reported that genetic background may impact behavioral responses in different tests. Specifically, anxiety evaluated by the elevated plus maze test differed between the three tested mouse strains (C57BL/6N, DBA/2, and FVB/N), while the exploratory behavior and activity in the open field was strain-independent (Eltokhi et al. 2020). Similarly, when hybrid CB6F1 mice were compared with one of their parental strains, C57BL/6, the hybrid genotype showed more anxiety-like behavior (Corder et al. 2023).

Since in our study *Tent2* KO mice exhibited decreased anxiety, we decided to check if they will show differences in contextual fear memory acquisition and extinction, that was not studied before (Fig. 4K). We noticed an increase in locomotion (low freezing level) in *Tent2* KO mice before the training (*P*-value = 0.01). This difference disappeared after delivery of three electric shocks (3US). There was no difference between WT and *Tent2* KO mice in freezing frequency during short-term (STM) and long-term memory test (EXT 24 h), indicating no impairments in formation and expression of short- and long-term memory. Moreover, when animals were exposed to the training context for 15 min without US presentation for contextual fear memory extinction, both WT and *Tent2* KO mice decreased freezing levels within the session and no difference between the genotypes were observed (*P*-value = 0.009, *P*-value = 0.039, *P*-value = 0.012). However, when fear extinction memory was tested on the next day, freezing levels were higher in WT mice, indicating that fear memory extinction was increased in *Tent2* KO mice.

### Decreased level of GABA in the hippocampus of *Tent2* KO

As we found changes in anxiety and fear extinction memory in *Tent2* KO mice, alterations in E/I balance as well as downregulation of transcripts involved in neurotransmitter transport and synaptic transmission, we decided to measure the concentration of amino acids and neurotransmitters in the hippocampus of *Tent2* KO and WT mice. After the last fear extinction test the level of GABA, a principal inhibitory neurotransmitter in the central nervous system, was significantly reduced (*P*-value = 0.0038) in *Tent2* KO (Fig. 4L). Moreover, concentration of glutamine, that is a precursor for glutamate, was increased (*P*-value = 0.0267). Although we did not observe statistically significant differences in the glutamate levels themselves, there was a trend toward its increased levels. These data align with decreased GABA-ergic neurotransmission and increased excitability in *Tent2* KO neurons (Fig. 3).

Our data suggest that the E/I balance is disturbed in Tent2 KO mice. Based on electrophysiological measurements, we observed increased intrinsic membrane excitability with no alteration in membrane potential or resistance that could explain this observation. Therefore, we focused our attention on other factors that may contribute to the alternation of E/I balance and we observed a significant increase in miniature excitatory and a decrease in inhibitory postsynaptic currents. Similar results were observed in a mouse model of tuberous sclerosis (TSC1 deficiency), where hyperactivity of the neuronal network was caused by a decrease in the amplitude and frequency of inhibitory miniature currents (Bateup et al. 2013).

Functioning of both principal excitatory neurons as well as inhibitory interneurons is crucial for maintaining the physiological balance of excitatory and inhibitory neurotransmission in the brain. Consequently, the dysregulation of E/I balance is a common feature of different neurological and neuropsychiatric disorders such as anxiety (Kalueff and Nutt 2007), schizophrenia (Nguyen et al. 2014; Heckers and Konradi 2015), bipolar disorder (Brady et al. 2013), and autism spectrum disorder (ASD) (Pizzarelli and Cherubini 2011). Actually, one of the ASD proposed mechanisms is the E/I imbalance theory. According to this theory, reduced inhibition in the cortex and hippocampus leads to over-stimulation of neurons and less efficient information processing (Rubenstein and Merzenich 2003; Sohal and Rubenstein 2019). Reduced inhibition was also reported in Mecp2 KO mice, a model of Rett syndrome and ASD. GABAergic neurons lacking MECP2 exhibit a decrease in GABA release and reduced level of mRNAs encoding GAD65 and 67 (Chao et al. 2010). In our *Tent2* KO mice, we detected decreased amplitude and increased inter-event intervals when the mIPSCs were measured, which indicates reduced GABAA receptor content per synapse and a lower number of inhibitory synapses. The level of GABA, the main inhibitory neurotransmitter in the CNS, was significantly reduced in *Tent2* KO mice after fear conditioning. Our data support the role of hippocampal GABAergic circuits in the regulation of fear acquisition (Oh and Han 2020). Abnormal hippocampal disinhibition may lead to attentional and memory deficits and result in disruptive consequences (McGarrity et al. 2017).

Posttranscriptional modifications of mRNA and microRNA molecules add a layer of complexity to gene expression, enabling context-specific regulation of translation, which is particularly crucial in neurons. Over two decades ago, it was hypothesized that cytoplasmic polyadenylation of synaptic mRNAs could be a major regulatory mechanism of local protein synthesis at synapses. The invertebrate cytoplasmic PAP TENT2 was shown to play a role in synaptic plasticity (Kwak et al. 2008). In mammals, its ortholog TENT2 (GLD2) has been proposed as a possible candidate for this function. However, there have been no reported brain-related physiological phenotypes of *Tent2* KO to date. Instead, the primary molecular phenotypes are associated with miRNA tailing rather than mRNA polyadenylation (Mansur et al. 2016, 2021), despite its ubiquitous expression in the brain. Here we show that dysregulation of TENT2 RNA targets results in the imbalance in the inhibitory and excitatory neurotransmission manifested by increased excitability of *Tent2* KO neurons and has an effect on animal behavior. Our data thus establishes a link between TENT2-dependent microRNA tailing and the balance of inhibitory and excitatory neurotransmission.

## MATERIALS AND METHODS

### Generation of *Tent2* knockout mice

The *Tent2* KO mouse line in C57BL/6/Tar x CBA/Tar mixed background was established using the CRISPR/Cas9 method in the Mouse Genome Engineering Facility (www.crisprmice.eu). The insertion of a 21 bp cassette in exon 3 (insTAATAGTGAggatccCTAGAT after p.Leu86) introduces 3xSTOP codons in the reading frame of the *Tent2* gene, as well as 1× STOP codons in +1 and +2 reading frames (Fig. 1A). Additionally, a unique BamHI restriction site for rapid and cost-effective genotyping was introduced in the insert. Based on the mouse genome (GRCm38/mm10 Assembly), a single guide RNA (sgRNA) was designed using an online CRISPR tool (http://crispr.mit.edu) and ordered from Synthego. The chosen sequence did not show any major off-targets but had high calculated efficiency.

Donor mice (B6CBAF1 Tar; C57BL/6/Tar x CBA/Tar) were injected first with 10 IU of PMSG (Pregnant Mare Serum Gonadotropin; Folligon, Intervet), and 48 h later with 10 IU of hCG (Human Chorionic Gonadotropin; Chorulon, Intervet) to induce superovulation. Females were mated with males (B6CBAF1 Tar) immediately after hCG injection. Zygotes were collected from mated females 21–22 h post hCG injection. Zygotes were microinjected into the cytoplasm using an Eppendorf 5242 microinjector (Eppendorf-Netheler-Hinz GmbH) and Eppendorf Femtotips II capillaries with the following CRISPR cocktail: Cas9 mRNA IVT (25 ng/μL), sgRNA Tent2_KO IVT (12.5 ng/μL), oligo_Tent2_KO (1.33 pM). After ∼2 h after microinjection, culture microinjected zygotes were transferred into the oviducts of 0.5 day p.c. pseudo-pregnant females. Pups from the F0 generation were genotyped at around 4 weeks. The presence of insertion was confirmed by sequencing in the founder mouse and, after backcrossing, in N1 generation mice. All mice were bred and maintained in the animal house of the Faculty of Biology, University of Warsaw under a 12 h light–dark cycle with food and water available ad libitum. The animals were treated in accordance with the EU Directive 2010/63/EU for animal experiments.

### Genotyping

Pups were genotyped at 4 weeks of age. DNA from tail or ear tips was isolated with Genomic Mini kit (A&A Biotechnology). gDNA was amplified with Tent2_Seq1F/1R primer pair using Phusion HSII polymerase and HF buffer.

PCR cycling:

- 98°C – 3:30
- 35× 98°C – 14 sec
  63°C – 17 sec
  72°C – 8 sec
- 72°C – 5:00

Primers used in the study:

- Tent2_Seq1F: 5′ ATGTGGTGACTCATTTTGGTAG 3′
- Tent2_Seq1R: 5′ TAGAAAATTAGGTACTCCTGATCC 3′

oligo_Tent2_KO (ssDNA repair template):

- atttgcttgttttcagGAGAATAAGCGATGAAAAAGCTTTTCCTCTTtaatagtgaGGATCCCTagAtgaGATGGTAAACGGCAGCGTTTCCATTCACCCCACCAAGAGCCAACTATAAT

### RNA isolation

Young adult male mice (6.5 week old) were sacrificed and the hippocampi were dissected. Total RNA was extracted using RNeasy Lipid Tissue Mini Kit (Qiagen). Next, 7 µg of total RNA from each sample was purified using KAPA Pure Beads (Roche), according to the manufacturer's instructions, and the quality and integrity of RNA was assessed on Agilent Bioanalyzer 2100 using Agilent RNA 6000 Pico Kit (Agilent 5067-1513). Purified RNA was further used for downstream analysis for total RNA sequencing and nanopore DRS.

### Library preparation and total RNA sequencing

RNA was ribodepleted and the libraries were prepared using KAPA RNA HyperPrep Kit with RiboErase (KAPA Biosystems, 08098140702), according to the manufacturer's recommendations. For library preparation, 1 µg of RNA was used, fragmented by 5 min incubation at 94°C. The library was enriched with nine amplification cycles. The quality of the enriched library was verified using High Sensitivity D1000 Reagents (Agilent 5067-5585) on Agilent TapeStation 2200. The libraries’ concentration was estimated by qPCR means with a KAPA Library Quantification Kit (Kapa Biosciences KK4824), according to manufacturer's instructions. These libraries were subsequently sequenced using an Illumina NovaSeq 6000 sequencing platform and NovaSeq 6000 SP Reagent Kit v1.5 (200 cycles) (Illumina 20040719) in 2 × 100 nt pair-end mode with standard procedure according to manufacturer's instructions, with 1% control library Phix (Illumina FC-110-3001).

### RNA-seq data analysis

Obtained reads were adapter-clipped and quality-filtered with Atria (4.0.0) (Chuan et al. 2021), then mapped to the reference mouse genome (GRCm39) with the STAR (Spliced Transcripts Alignment to a Reference) aligner (2.7.10a) (Dobin et al. 2013). Read counts were assigned to genes using featureCounts from the Subread (Liao et al. 2014) package (2.0.6) with Gencode vM26 annotation file using default parameters. Multimappers and reads overlapping multiple features were excluded. Differential expression analysis was done with DESeq2 (1.34.0) (Love et al. 2014) with default settings. Fold change was corrected using apeglm algorithm (Zhu et al. 2019). GO-term analysis was performed in R (4.1.2) environment using ClusterProfiler (Wu et al. 2021). Alternative polyadenylation (APA) analysis was performed in APAlyzer (1.9.4) (Wang and Tian 2020) with read cutoff for aUTR and cUTR equal to 5, *P*-value calculated through the unpaired *t*-test and corrected for multiple comparisons by the FDR method. Relative expression differences between PAS used in downstream data processing were represented by a relative expression score calculated with APAlyzer APAdiff() function. The results of the analyses were visualized using ggplot2 package (Wickham 2016).

### Nanopore direct RNA sequencing

Direct RNA-seq was performed as described by Bilska et al. (2020). Briefly, the 4.5–5 μg of total murine RNA was mixed with 150–200 ng of oligo(dT)25-enriched mRNA from *Saccharomyces cerevisiae* yeast, and spiked with 5 ng of in vitro transcribed poly(A) standards. Sequencing libraries were prepared with a Direct RNA Sequencing Kit (Oxford Nanopore Technologies, SQK-RNA002) according to the manufacturer's instructions. Sequencing was performed on a MinION device equipped with FLO-MIN106 (R9.4.1 RevD) flow cells and controlled with MinKNOW software (Oxford Nanopore Technologies). Base-calling was performed with Guppy 6.0.0 (ONT).

### Poly(A) tail length profiling

Reads were mapped to the Gencode vM26 (GRCm39) reference transcriptome with minimap2 2.17 (Li 2018) (-k 14 -ax map-ont ‐‐secondary = no) and processed with SAMtools 1.13 (Danecek et al. 2021) to exclude supplementary alignments and reads mapping to the reverse strand (SAMtools view -b -F 2320). The lengths of the poly(A) tails were estimated using the Nanopolish 0.13.2 polya function (Workman et al. 2019). In downstream analyses, only reads tagged by Nanopolish as “PASS” and “SUFFCLIP” were included. Statistical inference was performed using functions from the NanoTail R package (Krawczyk et al. 2024). The Wilcoxon signed-rank test was used to compare poly(A) length distributions across experimental conditions. Transcripts with a low number of supporting reads under each condition (<10) were excluded. *P*-values were adjusted for multiple comparisons using the Benjamini–Hochberg method. Transcripts were considered to have a significant change in poly(A) tail length if the length difference between the conditions was ≥5 nt and adjusted *P*-value was <0.05. GO-term analysis was performed in R (4.1.2) environment using ClusterProfiler (Wu et al. 2021). The results were visualized using the ggplot2 package (Wickham 2016).

### miRNA isolation

Adult male mice (3 weeks old) were sacrificed, and the hippocampi were dissected. miRNA was extracted using the Direct-zol RNA MiniPrep kit (Zymo Research R2050), according to the manufacturer instructions. The RNA quality was verified by Agilent Bioanalyzer 2100 with the RNA 6000 Pico Kit (Agilent 5067-1513). miRNA-seq libraries were prepared using the TruSeq Small RNA Library Prep Kit (Illumina RS-200-0036). The size distribution of the final libraries was validated with TapeStation 2200 using High Sensitivity D1000 Reagents (Agilent 5067-5585). Libraries’ concentration was determined using qPCR with the Kapa Library Quantification kit (Kapa Biosciences KK4824). For all above procedures, manufacturer's protocols were used.

### miRNA sequencing

The sequencing was performed using Illumina NovaSeq 6000 with the NovaSeq 6000 SP Reagent Kit v1.5 (200 cycles) (Illumina 20040719), generating 2 × 100 pair-end reads using the manufacturer's standard protocols with 1% addition of the control library Phix (Illumina FC-110-3001).

Raw sequencing reads were trimmed to remove excessive adapter and sequencing primer sequences using the BBDuk, a part of BBTools (BBMap – Bushnell B. – sourceforge.net/projects/bbmap/). Paired reads for each sample were processed together and specific parameters were applied (k = 23 mink = 11 hdist = 1 ktrim = r minlen = 17 tpe). For miRNA expression levels, only R1 reads were used. The miRNA expression levels and composition of the 3′ end of isomiRs were analyzed using QuagmiR (Bofill-De Ros et al. 2019). The QuagmiR was applied with searching for 5′ end variation filtering turned off and with specific custom parameters (min_ratio: .1 min_read: 9 edit_distance_3p: 3). Mature miRNA sequences, primiRNA sequences, and annotations of Mus musculus were from miRBase v22.

In the downstream analysis, we classified isomiRs as: (i) canonical (fully complementary to annotated mature miRNA sequence), (ii) trimmed (shorter than the annotated mature miRNA sequence), (iii) nontemplated (NT) tail (longer than the annotated mature miRNA sequence and tail sequence does not match genomic sequence), (iv) ambiguous tail (longer than the annotated mature miRNA sequence and tail sequence match genomic sequence, or shorter than the annotated mature miRNA sequence and tail sequence does not match genomic sequence). NT-tail was also analyzed in terms of tail length and specific sequences focusing on tails containing mono or combinations of adenosines and uridines.

All statistical analysis of isomiR classes and NT tail were performed with R custom scripts. The Wilcoxon test was used to determine *P*-values, and values <0.05 were considered statistically significant. The differential expression of isomiRs was performed with use of the DESeq2 R package (Love et al. 2014) with default settings. As cutoff for significant differentially expressed isomiRs, a threshold of adj. *P*-value <0.05 and |log_2_(fold change)| > 1 was set. All visualizations were performed with the ggplot2 R package (Wickham 2016). The miRNA expressions’ differential analysis results and isomiR composition can be found in Supplemental Data 2.

### Correlation of expression profiles between miRNA and mRNAs targets

The miRBase database target predictions implemented in the microRNA R package (Gentleman and Falcon 2024) were used to identify the miRNA and targeted mRNA pairs. miRNAs and their target mRNAs Ensembl IDs were initially filtered with a list of 158 miRNAs that were identified as having mono(A)-tailed isoform, as described above. For the investigation of expression profile's correlations, we focused only on significantly expressed genes. Thus, from initial pairs we analyzed 61 interactions with 22 miRNAs and 36 genes. For the analysis of correlation, we were comparing changes in expression measured as log_2_ fold changes as well as calculating Pearson correlation coefficient based on normalized reads. The annotation of genes with the process which it may potentially contribute to were based on the GO enrichment analysis combining filtering of the most enriched term with reduction of GO term, based on their semantic similarity and scores using the function reduceSimMatrix from rrvgo R package (Sayols 2023). The results are gathered in the form of heatmaps presented in figures, as well as in Supplemental Data 4.

### Patch clamp electrophysiology in acute brain tissue slices

Three to five week old mice were anesthetized with Isoflurane and decapitated. Brains were isolated and transferred into ice-cold cutting artificial cerebrospinal fluid (ACSF) containing 87 mM NaCl, 2.5 mM KCl, 1.25 mM NaH_2_PO_4_, 25 mM NaHCO_3_, 0.5 mM CaCl_2_, 7 mM MgSO_4_, 20 mM d-glucose, and 75 mM sacharose equilibrated with carbogen (5% CO_2_/95% O_2_). The brain was cut into two hemispheres, and 350 μm thick horizontal brain slices were cut in ice-cold cutting ACSF on a 5100mz vibratome (Campden Instruments). Slices were then incubated for 15 min in cutting ACSF at 32°C. Next, the slices were transferred to recording ACSF containing: 125 mM NaCl, 2.5 mM KCl, 1.25 mM NaH_2_PO_4_, 25 mM NaHCO_3_, 2.5 mM CaCl_2_, 1.5 mM MgSO_4_, 20 mM d-glucose equilibrated with carbogen and incubated for a minimum 1 h at RT. Patch-clamp recordings were recorded in a submerged chamber perfused with recording ACSF at 3 mL/min in RT. Patch pipettes (resistance 4–6 MΩ) were pulled from borosilicate glass (WPI, 1B120F-4) with a micropipette puller (Sutter Instruments, P-1000) and filled with internal solution containing 95 mM potassium gluconate, 6 mM KCl, 2 mM NaCl, 0.5 mM EGTA, 20 mM HEPES, 4 mM MgATP, 0.4 mM NaGTP, and 10 mM Na_2_ phosphocreatine (pH 7.4). For each slice, the measurements were done in stratum pyramidale of CA1. The firing pattern was recorded in the current-clamp mode by injection of depolarizing current stimulus. Recordings were acquired and digitized with a dual IPA amplifier (Sutter Instruments) and analyzed with Clampfit 10.7 software (Molecular Devices) and AxoGraph 1.7.4 software (developed by Dr. John Clements).

### Patch-clamp electrophysiology in primary hippocampal neuronal cultures

Whole-cell patch-clamp recordings in voltage-clamp mode were performed in 21–22 DIV in hippocampal cultures using borosilicate patch pipettes (4–6 MΩ) filled with one of the intracellular solutions described below. The external solution contained 120 mM NaCl, 5 mM KCl, 2 mM CaCl_2_, 1 mM MgCl_2_, 20 mM glucose, and 10 mM HEPES (pH 7.4, adjusted with NaOH). The mEPSCs were recorded at a holding potential of −60 mV after bath application of 10 μM gabazine and 1 μM tetrodotoxin (TTX) . The internal solution contained 95 mM potassium gluconate, 6 mM KCl, 2 mM NaCl, 0.5 mM EGTA, 20 mM HEPES, 4 mM MgATP, 0.4 mM NaGTP, and 10 mM Na_2_ phosphocreatine (pH 7.4). At the end of recording, 20 μM AMPA/kainate receptor antagonist 6,7-dinitroquinoxaline-2,3-dione (DNQX) was used to confirm the origin of the recorded mEPSCs. The mIPSCs were recorded at a holding potential of −60 mV in the presence of 1 μm TTX and 20 μM DNQX with an internal solution containing 140 mM CsCl, 2 mM NaCl, 10 mM HEPES, 5 mM EGTA, 2 mM MgCl_2_, and 2 mM Na_2_-ATP (pH 7.4). After the end of recording, 10 μM GABAA receptor antagonist gabazine was used to confirm the origin of the recorded mIPSCs. Extracellular solution was perfused at 3 mL/min. All recorded signals were low-pass filtered at 5 kHz, using the filter built into the Dual IPA patch-clamp amplifier, digitized at 20 kHz (Dual IPA amplifier, Sutter Instruments), and acquired with the SutterPatch software (Sutter Instruments). The series resistance (*R*_s_) was estimated from the response to a hyperpolarizing voltage step (−5 mV). The recordings in which series resistance was >20 MΩ were rejected. All electrophysiological data were analyzed with Clampfit 10.7 software (Molecular Devices) and AxoGraph 1.7.4 software (developed by Dr. John Clements).

### Behavioral assays

Adult male WT and *Tent2* KO mice were used. Experiments were performed sequentially in the following order: marble burying, elevated plus maze, open field test. A cohort of: WT, *n* = 9 and KO, *n* = 8 mice was used. The contextual fear conditioning was performed with another three separate cohorts of animals (WT *n* = 4, *n* = 7, *n* = 11 and KO *n* = 4, *n* = 6, *n* = 8). Mice were habituated to the room and the experimenter; assays were conducted during the light phase of the light–dark cycle. All the apparatus used were thoroughly cleaned with 70% ethanol between animals. Mouse behavior was video recorded and analyzed using EthoVision XT (Noldus).

### Marble burying

The test was conducted in a polypropylene box (43 cm length, 26 cm width, 31 cm height) filled with 5 cm depth of fresh bedding. Animals were habituated to the box 2 days for ∼1 h each day. On the third day, 15 clear glass marbles (2 cm diameter) were placed evenly spaced in three columns on the surface of the bedding. Animals were placed separately onto the central area of the box and allowed to freely explore the new environment. After 30 min of testing, mice were placed back to their home-cage and the number of marbles that were buried to two-thirds of their depth were counted manually. Whole experiments were videotaped by the overhead mounted camera and analyzed with the EthoVision XT tracking system (Noldus). Each mouse was given fresh bedding and marbles were previously cleaned with 70% ethanol and water to eliminate any olfactory cues. All experiments were performed in darkened lighting conditions (4–5 lux).

### Elevated plus maze

Testing was performed in a metal plus-maze apparatus elevated to a height of 50 cm (AnimaViVari). The apparatus consisted of two open arms and two closed arms, both of the same dimensions (35 × 5 cm), with gray PCV walls 15 cm high and arranged so that both the open and closed arms faced each other. The testing was done in a dimly lit (30 Lx at the maze level) area surrounded with nontransparent, gray curtains in order to limit any additional spatial stimuli. Mice were placed at the intersection of the open and closed arms, and mouse behavior was recorded by camera during a 5 min test interval and analyzed with the EthoVision XT tracking system (Noldus). The following parameters were calculated in open and closed arms: number of entries, total time spent, distance moved and movement duration; in the central platform: time spent; in the whole arena: total distance moved, total movement duration.

### Open field test

Testing was performed in a plexiglass box (dimensions 64 × 64, height 32 cm). Mice were placed in the middle of the arena and animal's movements were recorded for 15 min by a camera set up above the arena. The video was analyzed using an EthoVision XT video-tracking system (Noldus) to extract behavioral data. Two zones, the center zone (inner 50 cm diameter circle) and the border zone (remaining 6 cm border), were defined according to previously obtained criteria. The following parameters were taken into analysis—time spent, distance moved, and movement duration. The arena was cleaned after each animal with a 70% solution of ethanol and dried.

### Contextual fear conditioning

Mice were trained in a Med Associates Inc. Fear Conditioning Chamber connected to a computer running Video Freeze software. Thirty minutes before training, mice were brought to the room with the conditioning chamber to acclimatize. Chambers were cleaned with 70% ethanol and the paper towels soaked with ethanol were placed under the metal grid. Mice were placed in the chamber on a metal grid platform and after 148 sec of habituation, received three electric shocks (US, 2 sec, 0.7 mA) with 90 sec intervals—the training lasted 6 min. The animals were taken out from the experimental chamber 30 sec after the last shock and put in the home-cage. Between animals, the cage was cleaned with 70% ethanol solution and a fresh paper towel was placed. The fear memory of the context, defined as a level of freezing in the context, was assessed for 5 min in the same experimental chamber 1, 24, and 48 h after training. All animals were trained, tested, and sacrificed during the light phase of the animals’ day (between 09.00 and 16.00 h). The training and testing times were counterbalanced between the groups. Animals were sacrificed after the last test session, hippocampi were dissected and frozen on dry ice.

### Quantitative determination of amino acid profile

Frozen hippocampi were lyophilized using an Alpha 2-4 LD Plus lyophilizer (Martin Christ). Amino acid levels were measured using high-performance liquid chromatography coupled with tandem mass spectrometry (LC-MS/MS) in a commercial laboratory (Masdiag).

## DATA DEPOSITION

The raw sequencing data (FAST5 files) were deposited at the European Nucleotide Archive (ENA) (https://www.ebi.ac.uk/ena/browser/home) under project numbers PRJEB76814 and PRJEB75356. The RNA-seq data were deposited in ENA under project number PRJEB76814. The miRNA-seq data were deposited in ENA under project number PRJEB76999. All sample IDs, accession numbers, and other metadata are provided in Supplemental Data 1.

## SUPPLEMENTAL MATERIAL

Supplemental material is available for this article.

## Figures and Tables

**Patrycja Wardaszka-Pianka:**
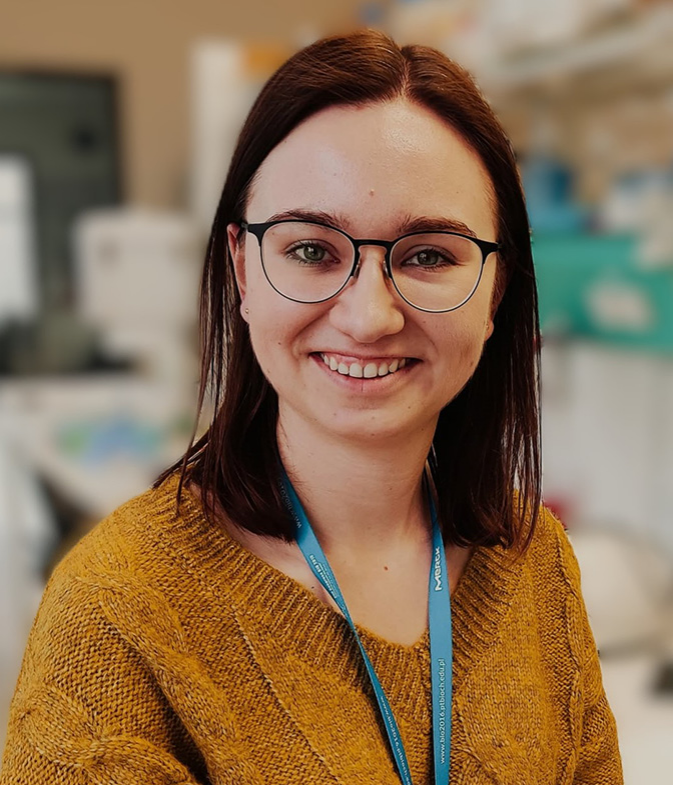


## MEET THE FIRST AUTHOR

***Meet the First Author(s)* is an editorial feature within *RNA*, in which the first author(s) of research-based papers in each issue have the opportunity to introduce themselves and their work to readers of *RNA* and the RNA research community. Patrycja Wardaszka-Pianka is the first author of this paper, “Terminal nucleotidyltransferase *Tent2* microRNA A-tailing enzyme regulates excitatory/inhibitory balance in the hippocampus.” Patrycja is a PhD student at the Faculty of Biology, University of Warsaw in Poland. She is particularly interested in RNA tailing in neurons.**

**What are the major results described in your paper and how do they impact this branch of the field?**

The major results described in this publication relate to changes in RNA tailing in mouse neurons lacking the terminal nucleotidyltransferase TENT2. We observed that miRNA monoadenylation is altered in the absence of TENT2 without global changes in the level of mRNA polyadenylation. However, differential expression analysis showed that neurotransmission-related mRNAs are downregulated in *Tent2* KO. These molecular changes translated into behavioral abnormalities such as reduced anxiety and improved fear memory extinction, as well as changes in electrophysiological properties of neurons, an imbalance of excitation and inhibition. Our work extends knowledge of the role of TENT2 in neurons. We show that it is different than during gametogenesis, where it is responsible for mRNA polyadenylation. In neurons, TENT2 affects transcripts more indirectly—through miRNAs.

**What led you to study RNA or this aspect of RNA science?**

As a student, I attended a course on RNA analysis techniques. I was particularly interested in a class on “RNA study in neurons,” where I learned about the concept of cytoplasmic polyadenylation, which can regulate local translation at synapses. This point seemed particularly interesting to me. When I had the chance to join a project on cytoplasmic polyadenylation in neurons, I was very happy to take it.

**During the course of these experiments, were there any surprising results or particular difficulties that altered your thinking and subsequent focus?**

The biggest surprise was the results of direct RNA sequencing when we found that, despite the absence of the TENT2 enzyme, the total length of the poly(A) tails of the transcripts remained unchanged. We expected it to be the opposite, since in early development TENT2 is the main enzyme responsible for polyadenylation of cytoplasmic mRNA. We realized that it is different in neurons. TENT2 primarily regulates tailing of specific miRNAs.

**What are some of the landmark moments that provoked your interest in science or your development as a scientist?**

I believe that the landmark moments which influenced my development as a scientist include internships and participation in international conferences. The opportunity to confront my results with the opinions of other scientists is incredibly valuable—this is where I receive constructive feedback, suggestions, and questions that challenge me and inspire further growth. Meeting scientists who are passionate and deeply engaged in their work is highly motivating and encourages me to continue exploring and expanding my knowledge.
